# A Mixed Method Approach for the Investigation of Consumer Responses to Sheepmeat and Beef

**DOI:** 10.3390/foods9020126

**Published:** 2020-01-24

**Authors:** Melindee Hastie, Hollis Ashman, Damir Torrico, Minh Ha, Robyn Warner

**Affiliations:** School of Agriculture and Food, Faculty of Veterinary and Agricultural Sciences, The University of Melbourne, Melbourne, VIC 3010, Australia; hollis.ashman@unimelb.edu.au (H.A.); Damir.Torrico@lincoln.ac.nz (D.T.); minh.ha@unimelb.edu.au (M.H.); robyn.warner@unimelb.edu.au (R.W.)

**Keywords:** consumers, attitude, sensory, beef, sheepmeat, premium, holistic product development

## Abstract

Coupling qualitative and quantitative consumer research methodologies enables the development of more holistic and comprehensive perspectives of consumer responses. In this study, consumer responses to beef and sheepmeat were investigated using a mixed method approach combining perceptual mapping (qualitative), and sensory (quantitative) methodologies. Qualitative insights indicated Australian and Asian consumers differ in perception of familiarity and ‘premiumness’ of meat products. Specific findings included: Australians consume grilled or roasted meat as a centre of the plate ‘hero’ ingredient, while Asians prefer stovetop cooking methods where meat is one ingredient in a complex dish. Labelling meat as ‘Australian’ was important for Australian consumers but not for Asian consumers. Quantitative data demonstrated that older consumers (31–70 years) scored sheepmeat higher than younger consumers (18–30 years) for healthiness (*p* = 0.004), juiciness (*p* = 0.029), odour liking (*p* = 0.005) and tenderness (*p* = 0.042). Older consumers also had a lower willingness to pay than younger consumers for “premium” quality meat; 30–40 vs. 40–50 AUD (Australian dollar) per kg respectively for sheepmeat, and 40–50 vs. 50–60 AUD per kg respectively for beef. In conclusion, the approach used effectively integrated consumer attitudes, usage information and sensory assessments with socio-demographic factors to generate insights for the refinement of market strategies and product offerings.

## 1. Introduction

Australia, one of the countries with the highest meat consumption rate per capita in the world, has experienced a steady decline in red meat consumption over the past 30 years, with beef consumption dropping from 39 to 26 kg/person/year and sheepmeat from 25 to 8.5 kg/person/year [1]. The decline has been linked to consumers reducing their red meat intake in response to health warnings and the increasing affordability of alternative protein sources [1].

Australia now exports more red meat than the local market consumes [2,3], and the nearby developing markets of Asia are increasingly important for the Australian red meat industry. Australia is not alone in seeking to maximise its Asian export opportunity, and competes with other exporting nations such as Brazil, India, USA and New Zealand for a share of this market [3,4]. In the commoditised global red meat market, Australia is less competitive on a price basis; thus, “premiumisation” of Australian beef, sheepmeat and pork products is currently employed as a key strategy for market differentiation and enables access to the growing numbers of affluent and discerning consumers in both Asia and Australia, facilitating realisation of higher financial returns [2,3,4,5].

However, there is evidence of cultural differences in consumer responses to red meat. Simply “lifting and launching” products, and marketing formats that work in Australia and applying them in the Asian market, may not be maximising the export opportunity [6,7,8,9]. Consumers, ultimately, are the purchase decision makers. Understanding their attitudes to, and perceptions of, red meat along with the attributes linked to consumer choice and “premiumness” can inform the development of more targeted marketing strategies and optimised product development, thereby maximising the opportunity for Australian red meat products in a highly competitive environment [10,11,12,13,14].

Consumer responses to red meat are influenced by a complex range of dynamic, interrelated factors. Font-I-Furnols and Guerrero [15], in referring to consumers’ responses to pork, categorised these factors as: (1) product’s sensory qualities; (2) marketing factors (information received by the consumer in the form of advertising, labels, etc.); and (3) psychological factors (the consumer’s motivations, expectations and perceptions). However, most existing research tends to be isolated and fragmented, residing either within the consumer science realm (focused on the attitudes or purchase decision drivers), or the sensory science realm (focused on the consumption experience) [16,17,18]. Combining methodologies from these two realms allows the researcher to develop a more holistic and detailed view of the consumer’s response and determine if modifications of the current product offering are required to increase its appeal to the target consumer, as previously done for pork by Bredahl et al. [14], and Dransfield et al. [19].

The overall objective of this study was to explore the effectiveness of a “mixed method” approach to investigate both local and export market consumer responses and attitudes towards beef and sheepmeat attributes, and to understand how they might differ for consumer groups according to their sociodemographic features. Revealing these differences will inform the development of differentiated marketing and product design for Australian red meat in accordance with target consumers’ preferences.

## 2. Materials and Methods

This study combines:

(a) a qualitative analysis of consumers’ attitudes and perceptions through facilitated discussions and “perceptual mapping exercises” as described by Beckley et al. [20], Nestrud and Lawless [21] and Risvik et al. [22], with

(b) a quantitative sensory assessment of eating quality based on MSA (Meat Standards Australia) sensory protocols as described by Watson et al. [23] and, subsequently, a priori consumer segmentation analysis based on demographic factors, a method previously described by Dolnicar et al. [24].

All subjects gave their informed consent for inclusion before they participated in the study. The study was conducted in accordance with the Declaration of Helsinki, and the protocol was approved by the Human Research Ethics Committee of The University of Melbourne (HREC 1545786.1).

### 2.1. Overview of Qualitative Approach

Variables examined in the qualitative study included consumer cultural heritage (Asian or Australian) and meat species (beef or sheepmeat). Perceptual mapping exercises were completed by both consumer groups for both meat species separately. Each session focused on a defined set of stimuli samples according to an experimental design that encompassed a range of meat attributes, e.g., cut, colour, fat content, freshness (see Section 2.1.3). Additionally, at the end of the perceptual mapping sessions, participants for the sheepmeat sessions completed a sheepmeat descriptor mapping exercise (Section 2.1.5), and beef session participants completed a beef concept mapping exercise (see Section 2.1.6).

#### 2.1.1. Panels

Seven panels were run in total at the University of Melbourne. A total of 67 panelists were recruited from the university population. The group’s age ranged from 18–58 years and included 31 male participants and 36 female participants. Most participants were postgraduate students (84%) with ages ranging from 22–58 years. Panelists were asked to self-identify as Australian or Asian before being assigned to either a sheepmeat or beef panel. Asian ethnicity was defined as having been born in Asia and having been a resident in Australia for less than two years. Separate panels were run for Asian and Australian consumers, and these panels focused on either sheepmeat or beef. For sheepmeat, a total of 26 Australians participated in three sessions, and 18 Asians participated in two sessions. For beef, one session was run with six Australian participants and one session was run with 17 Asian participants. Each session was run for approximately 1.5 h and was led by a trained facilitator with at least one note taker/observer; sessions were also video recorded for later review.

#### 2.1.2. Discussion Guide and Facilitation

A discussion guide was used throughout the beef and sheepmeat panels (Supplementary A), providing the facilitator with timings and topics to be covered during the session. In brief, the sheepmeat discussion guide included: 5 min for the introduction of researchers and participants; 15 min for seeking information on familiarity with sheepmeat, frequency of consumption, eating occasions and understanding of the terms hogget, mutton, and lamb (In Australia, lamb is defined as an ovine animal under 12 months of age with no permanent incisors in wear; hogget is older than a lamb, has lost its milk teeth, can have up to two permanent teeth in wear and is usually between 12 months and 2 years of age; mutton is older again and will have more than two permanent incisors [25,26]); 45 min for a stimuli mapping exercise (see Section 2.1.4); 20 min target for sheepmeat descriptor mapping (see Section 2.1.5). 

A similar discussion guide structure was used for beef except that the final 20 min sheepmeat descriptor mapping exercise (see Section 2.1.5) was replaced with a beef concept mapping exercise (see Section 2.1.6). All facilitators were experienced with the perceptual mapping methodology, were aware not to use leading questions and ensured everyone within the group had the opportunity to provide their opinions on mapping decisions. They were also free to explore new topics if they arose during the discussion.

#### 2.1.3. Stimulus Selection

For each group, the participants were presented with the same set of either sheepmeat or beef images in a designated order. Images were sourced from the University of Melbourne’s collection or adapted from cited sources and selected to reflect the stimuli design of experiment to test the range of the sensory or product space. In brief, the images demonstrated a variety of retail and primal cuts from sheep and beef (including some familiar market cuts from Asia and some from Australia). All images were of uncooked meat on a plain white background printed in colour on a high gloss photo paper measuring 130 mm × 80 mm. The images were unlabelled, but participants were informed that all images were either beef, or sheepmeat according to the session. Images incorporated a range of meat colours (ranging from light pink to dark red, assessed visually to reflect the range of Australian retail meat) and ranged in freshness (freshly cut meat to slightly spoiled meat), with varying amounts of fat (both subcutaneous and IMF (intramuscular fat)) and varying amounts of preparation (e.g., untrimmed joint, stringed roasting joint, chops, deboned, French trimmed). The image/stimuli descriptions, design of experiment, personation order and source are detailed in Supplementary B.

#### 2.1.4. Perceptual Mapping

During the perceptual mapping exercise, consumers were asked to sort and place the set of stimulus samples onto a predetermined two-dimensional map according to their similarities and differences. To enable comparison between groups, the x and y dimensions of the map were labelled by the facilitator before group mapping commenced. The x-axis ranged from every day to premium and the y-axis from unfamiliar to familiar. The selection of these axes was based on the fact that “premiumisation” is a key strategy for the marketing of Australian products [2,3,4]. Familiarity was chosen as it is understood that consumer acceptance can be enhanced by product familiarity [27,28,29] and, conversely, unfamiliarity can be a barrier to consumer acceptance [30]. While the final map is informative, the discussion undertaken within the group during the exercise also generates insights into how the consumers assess product attributes, and it is from this discussion that most insights were distilled. Sheepmeat stimuli were used initially with replicate sessions to validate insight development, and beef sessions were subsequently used to compare/test those insights. Mapping was completed on a tabletop with the axis marked out using masking tape. For all sessions, the same first sample was placed by the facilitator at the centre of the map. For the sheepmeat sessions, the image selected was a leg of lamb and in the case of beef, porterhouse steak from the striploin. Both cuts were chosen to represent the middle of the range in terms of “premiumness” and are common formats in the Australian market. All subsequent samples were presented by the facilitator to be placed on the map by the group (one at a time and in predetermined order as described in Supplementary B).

#### 2.1.5. Sheepmeat Descriptor Mapping

At the end of the sheepmeat mapping session, all groups were presented with a random set of descriptors laid out on a tabletop, each printed individually on a plain white card of similar dimensions to the mapping stimulus used previously. The descriptors were derived from production and eating quality factors and were selected to cover a range of premiumness and familiarity for the two consumer groups and are described in detail below.

##### Production Descriptors

“Lamb”, “Spring lamb”, “Natural”, “Dry-aged”, “Australian”, “Lean”, “Organic”, “Healthy” and “Fresh” were selected from labels already in common use on the Australian and Chinese market and were expected to be familiar terms for both groups.

“Hogget” and “Mutton” are not in common use in Australia due to the fact that lamb is the main commercial source of sheepmeat; they are, however, important product categories for export [25,31], and therefore were included.

“Green” was selected as a familiar label for Asian participants as there are a number of established green label certification systems operating in Asia and there is evidence of Asian consumer preference for these labels [32,33,34].

“Mutton” is also a term often used in the Indian subcontinent to describe goat meat, and “Goat” was included to ensure consumers were differentiating goat from sheep mutton [35].

Other production terms included “Small portion” and “Scientific”.

##### Eating Quality Descriptors:

Eating quality descriptors from the literature included consumer-friendly terms describing sheepmeat; “Juicy”, “Succulent”, “Tasty”, “Fatty”, “Plain” “Sweet”, “Tender”, “Bitter”, “Crispy” and “Firm” [36,37,38]. In addition, “Neutral”, Hot” and “Cool” were included to represent traditional Asian food classification systems. [39,40,41]. 

In order to identify the most important descriptors for each of the consumer groups, participants were asked to select the top three descriptors they would like to see on their meat as a group. They were then asked to place these descriptors onto a large target image mounted on the wall in order of importance (the centre of the target being the most important product).

#### 2.1.6. Beef Concept Mapping

Concept phrases were developed to test a range of provenance factors with the aim of identifying the provenance attributes considered “premium” by the two consumer groups. The selected provenance (extrinsic) factors were developed from the descriptor mapping results for sheepmeat, and provenance stories already being used in the market for “premium” meat products. Concept phrases were printed individually onto photo paper cut to the same dimensions as the perceptual mapping exercise images. They were presented to the group one at a time in a prescribed order (randomised on the basis of statement complexity), and the group was asked to place these concepts onto the same axes used for the perceptual mapping exercise (Section 2.1.4). Concepts, presentation order and their associated provenance attributes are detailed in Table 1.

#### 2.1.7. Analysis of Results

At the end of each session, transcripts were typed up by the assigned note-takers and reviewed by the research team. To reduce bias in the analyses of session outputs, the facilitators, observers and note-takers met after each session, reviewed the final set of notes, and compared the maps from each of the sessions. Observations were discussed by the group, comparisons were made between sessions, and insights were generated and validated within the research team. Insights were then collated into themes and compared across the two cultural groups.

### 2.2. Quantitative Sensory Methodology

#### 2.2.1. Samples

Subsequent to the panels (at a later date and using different participants), a sensory experiment was conducted where consumers assessed the eating quality of either beef or sheepmeat. Two samples were selected for each group to provide a range of marbling (intramuscular fat) and ageing method characteristics. The samples were aged by either the conventional method of wet ageing (vacuum packed then stored in a chiller to age at 0.5–2.0 °C) or the “premium“ method of dry ageing—where unpackaged meat is hung in low temperature (2–4 °C), in a humidity controlled environment (RH approx. 85%) for a defined ageing period [42]. Beef samples used were: beef striploin (longissimus lumborum wet (*n* = 4) or dry-aged (*n* = 4) for 35 days. The wet-aged beef was MSA marble score 500 and the dry-aged beef was marble score 300 [43]. Sheepmeat samples included wet-aged boneless ‘lamb’ loins (longissimus thoracis et lumborum *n* = 6, wet-aged for 14 days) and bone in ‘mutton’ loins (longissimus thoracis et lumborum, *n* = 6, dry-aged for 35 days), from Dorper sheep. All samples except for the dry-aged mutton were sourced from a specialty butcher in Melbourne, Victoria, Australia. The dry-aged mutton was sourced from a specialist dry-ageing facility located in the Adelaide Hills district of South Australia, Australia. In addition, a ‘link’ (assumed mid-range eating quality sample served at the commencement of every tasting session in order to familiarise the consumer with the product and protocol, results not used) was purchased from a supermarket in Melbourne, Victoria, Australia. Beef link samples were wet-aged MSA graded beef porterhouse steaks (*n* = 10) fabricated from longissimus lumborum, and for sheepmeat, the link sample was wet-aged lamb mid-loin chops (*n* = 30) fabricated from longissimus thoracis et lumborum. All samples were received at the University of Melbourne meat research centre under refrigerated conditions the day before the sensory session. Upon receipt, all samples were prepared as boneless portions, ready for grilling at the sensory session. The sheepmeat link samples and dry-aged sheepmeat samples were deboned, and the subcutaneous fat was trimmed to a similar thickness for all samples. The deboned beef and sheepmeat samples were then cut into steaks of the same thickness (2.5 cm for beef and 1.5 cm for sheepmeat). The prepared sheepmeat and beef samples and link samples were then vacuum packed and kept refrigerated at 2 °C until required for testing.

#### 2.2.2. Consumers

Consumers (*n* = 75) were solicited as they walked past sensory booths. Upon consenting to participate in a tasting, they self-selected for either beef or sheepmeat. This self-selection strategy facilitates the participation of consumers familiar with the product being tested. Before commencing the sensory sessions, participants were provided with verbal and written instructions covering demographic survey completion, palate cleansing procedures, completion of the sensory assessment form and tasting procedures. In addition, the first tasting (a link sample, data not used) provided a training opportunity for those participants not experienced with the sensory methodology. Experienced staff checked paperwork throughout the sessions and were available to answer any participant queries. All participants completed a demographic survey based on the survey format of Hwang et al. [44] before sensory testing commenced.

#### 2.2.3. Sensory Testing

Up to four consumers participated in each sensory session, and each consumer tasted three samples. All sessions were started with a link sample and the sample presentation (wet-aged or dry-aged) was alternated between second and third order between sessions. Cooking method was based on the MSA protocols described previously by Watson et al. [23], Thompson et al. [45] and Polkinghorne et al. [46]. In brief, samples were grilled before serving, on a preheated silex clamshell grill with the top plate set to 185 °C and the bottom plate set to 195 °C. Samples were placed on the preheated grill, the top plate closed 30 s later, and grilled until they reached a medium level of doneness (approx. 3 min to reach an internal temperature of 60 °C). Once cooked, samples were rested for 2 min (while covered with foil), cut into equal size portions (a visually estimated 20 + 5 g, based on practice sessions carried out in the laboratory), then each portion was individually placed on a plate with foil placed over the meat and immediately served. All participants were provided with water crackers and 10% apple juice/water to cleanse their palate, before tasting commenced and between samples. Upon receiving the sample, consumers were asked to remove the foil and rate odour liking (Od) and then to taste the sample. While tasting, consumers recorded their response to tenderness (T), juiciness (J), flavour (F) and overall liking (OL) using the questionnaire format described in Watson et al. [23]. Consumers indicated their liking for each of the eating quality attributes by marking on 100 mm lines (score range = 0–100) anchored with the words ‘very tough/dry ‘to ‘very tender/juicy’ and ‘dislike’ to ‘like extremely’ for flavour/odour/overall liking. In addition to the eating quality assessments, consumers were also asked to rate each sample for health and premiumness by marking a similar scale anchored with words from ‘not healthy/premium’ to ‘very healthy/premium’. These marks were subsequently converted to an eating quality score out of 100 by measuring the distance of the mark from the point of origin of the scale in mm. After completing these assessments, they rated the quality grade of each sample by checking a box labelled “unsatisfactory”, “good everyday quality”, “better than everyday quality “or “premium quality”.

At the end of the sensory session, consumers were also asked to indicate how much they would be willing to pay (WTP) for each quality grade by selecting a price category from 0–10, 10–20, 20–30, 30–40, 40–50, 50–60, 60–70 or 70–80 AUD (Australian dollar) per kg. In addition, immediately after selecting a price category, consumers indicated likelihood to purchase the product (LTP) at the selected price point by marking a 100 mm scale anchored with words ‘not at all’ to ‘very likely’ (score range = 0–100).

The MSA eating quality scores of SEQ (sheepmeat eating quality score) and MQ4 (meat quality combined score for beef) were determined as described below according to Pethick [47] and MLA [48], respectively.

SEQ = 0.3 (T) + 0.1 (J) + 0.3 (F) + 0.3 (OL),

MQ4 = 0.3 (T) + 0.1 (J) + 0.3 (F) + 0.3 (OL),

where T = tenderness score out of 100, J = juiciness score out of 100, F = flavour liking score out of 100 and OL = overall liking score out of 100.

#### 2.2.4. Statistical Analysis

Statistical analyses of sensory test data were performed using REML in GenStat for Windows (16th Edition, VSN International, Hemel Hempstead, UK). Sheepmeat and beef data were analysed separately. The factors in the initial model were ageing method (wet or dry), and the random model included consumer ID. The effect of demographic data on sensory scores was investigated for each demographic factor. After visual inspection of the consumer age vs. sensory data using box plots (data not shown), two consumer clusters (differing according to their sensory scores) were identified. The data were then segmented into these two age categories being 18–30 years old (29% of data) and 31–70 years old (71% of data) to reflect the clusters and enable a comparison between younger and older consumers. The final parsimonious model for each species only included consumer age (18–30 years vs. 31–70 years) and the random model included consumer ID.

## 3. Results

### 3.1. Qualitative Results

#### 3.1.1. Perceptual Mapping of Sheepmeat and Beef

On reviewing the session transcripts and maps for sheepmeat, it was revealed that aside from the factors included in the experimental design, i.e., familiarity, premiumness, cut, colour, fat content, trimming, etc., there were a number of extrinsic factors, such as perceived tenderness, convenience, labelling, value for money and eating occasion, that consumers utilised when assessing the “premiumness” of a sheepmeat product. These factors, along with the design of experiment factors were captured as themes and are detailed in Supplementary C, Table SC1, along with notes on how each of the groups assessed the factors. Figure SC1 contains exemplars of the Australian and Asian consumer maps for sheepmeat. In general, the themes developed from the sheepmeat perceptual mapping exercise were also found in the beef perceptual mapping exercise, therefore the results were tabulated in a similar manner to enable comparison between the sessions (Table SC2 of Supplementary C). Figure SC2 provides exemplary maps from the beef perceptual mapping sessions for Australian and Asian consumer groups.

The perceptual mapping results were combined with the sheepmeat target exercise results and the beef concept results to generate the insights described in Section 3.3.

#### 3.1.2. Sheepmeat Descriptor Mapping Results

Table 2 summarises the results of the sheepmeat descriptor mapping. For the two Australian groups, “Australian” was the most important descriptor for the preferred sheepmeat product, demonstrating the importance of a product’s origin for Australian consumers. Group 1 then selected “Spring lamb” followed by “Tender/fresh” (they were not able to reach consensus on the third most important factor), while group 2 selected “Organic” followed by “Tasty”. Both Asian groups selected “Fresh” in their top three descriptors (most important for group 1 and second most important for group 2). They also selected “Tender”, “Tasty” and “Organic” and in common with the Australian groups, “Aged” was only selected by group 2 and when questioned whether a product could be aged and fresh, they were clear it could be; later conversations with these consumers revealed the term fresh was used as opposed to frozen.

#### 3.1.3. Concept Phrase Testing Results for Beef

The concept mapping results for beef are detailed in Supplementary C Table SC3. While both groups mapped “Fresh Australian Beef” into the familiar everyday quadrant, there were marked contrasts in how Asian and Australians mapped the remaining concepts. For the Asian group, all other concepts were unfamiliar but mapped as premium. For Australians, the deliberately unfamiliar concept “Traditional Australian breeds like Brahman or Angus” was mapped into the unfamiliar everyday quadrant, but the remaining concepts were mapped in the familiar premium quadrant. It should also be noted that the mapping by Australian consumers was a slower process than for the Asian consumers, due to the amount of discussion in the Australian group regarding the validity of each claim.

### 3.2. Insights Generated from Qualitative Assessments of Sheepmeat and Beef

As hypothesised, it was found that Australians and Asians assess visual meat quality attributes and concepts differently; for instance, Australians rely very heavily on labelling/cut identification to assess a product’s eating quality, and they showed a preference for locally produced product and a degree of skepticism concerning the premiumness of unfamiliar cuts, most often mapping unfamiliar cuts to the “everyday” side of the map. The Asian group, on the other hand, were comfortable choosing meat for home use based on visual quality cues, mapping meat without labels relatively easily and plotting unfamiliar cuts as premium based only on visual quality cues.

The Australian requirement for labels could be attributed in part to home usage practices; during this study, it was found Australians tend to use meat as the centrepiece of a meal with a range of cooking methods utilised at home; e.g., high-temperature, short duration cooking methods such as grilling or BBQ, medium temperatures, medium time methods such as oven roasting and low temperature, long time cooking such as braising and slow cooking. This range of cooking methods generates different eating experiences with varying cuts of meat and getting it wrong could lead to an unsatisfactory eating experience. For instance, oven roasting beef round steak produces a very tough and probably inedible dish, so this cut is better suited to low temperature long time cooking methods. The fear of getting it wrong, combined with the common Australian retail practice of providing cooking instructions, has created a label dependency for this group. For the Asian group, purchase of meat was traditionally at the “wet market” and based on visual quality cues (not labels), and at home preparation typically involved incorporation of small pieces of meat into a complex dish, usually via a stovetop cooking method (e.g., stir fry or slow-cooked curry) with tenderness determined by format (such as thin slicing) and cooking time.

#### 3.2.1. The Channel for Premium

The differences in cooking styles for the two groups raised questions around premiumisation strategies. When meat is a centre of the plate “hero” ingredient, it is relatively easy to understand how a high-quality luxury eating experience might be delivered by a special piece of meat (e.g., dry-aged or wagyu). However, where meat is part of a complicated dish, how does the “premium” quality of the meat stand out? Inquiries into home vs. restaurant use highlighted that the channels for “premium” meat might differ for the two groups. For instance, Australians (at least those who are experienced home cooks) are comfortable purchasing premium meat from high end butchers and preparing it at home, especially for special occasions, whereas many Asians (not so familiar with Australian cooking styles) and those Australians who are not experienced cooks would prefer these premium eating experiences happen in the restaurant, (where professionals have taken on the responsibility of preparing these costly cuts of meat and created a whole environment for enjoying the eating experience). It was also noted that the butcher plays a special role in the selection of premium meat for Australian consumers, many describing discussions with the butcher where they seek advice on meat selection and the best way of preparing the meat at home.

#### 3.2.2. Labelling Requirements for Home Use and Retail

Australians’ strong preference for products labelled as “Australian” and their need for labelling to enable cut identification, both suggest labels identifying the cut and recommended cooking methods are a minimum requirement for Australian retail, and preference would be given by consumers to those products that are labelled “Australian”.

For Asian consumers, labelling was useful, but its absence was not a barrier to potential purchase. While it was clear that being labelled as Australian was important for Australian consumers, it was equally clear this was not as important to the Asian consumer. For this group, an “Australian” label would not drive the perception of premiumness. The unfamiliar beef concepts covering provenance factors of health, brand, uniqueness craftmanship and eating quality were all well received by this group and would enhance the premiumness of the meat. These results suggest there is considerable scope to leverage stories of provenance for the export of red meat from Australia to Asia. Australian consumers displayed a more skeptical attitude to the beef concepts, mapping familiar concepts to premium and unfamiliar concepts to everyday. From the group discussion, it was clear that claims needed to be validated by Australian consumers before acceptance.

#### 3.2.3. Response to Cut, Colour, Bone and Fat Content

The two groups placed different levels of importance on the way the meat was cut; Asians preferred to cut meat to the desired format at home while Australians preferred convenient cuts, such as mince, strips and cubes, for cooking on weeknights and special cuts for weekends and entertaining. The acceptability of bone content also differed between the groups, with Australians in general considering bone as waste, while Asians who selected meat cuts with bone were often planning to use the bone in soup or stock. There also appeared to be differences in what was considered acceptable fat content for the two groups, with Australians expressing a preference for leaner meat and actively avoiding visible fat for health reasons. The Asian group differentiated subcutaneous fat and intramuscular fat (IMF), and too much subcutaneous fat was undesirable but high IMF content was related to a premium eating experience.

The Asian consumers appeared more sensitive to the colour of the stimuli images than the Australian consumers, and they used it as an indicator of meat quality. Dark brick red meat was described as organic and fresh, while grey or pale tones indicated the meat was not fresh. From the sheepmeat descriptor mapping results (Table 2), freshness was identified as an important attribute for Asians, and the term fresh was used in contrast to frozen. Interestingly, the Asian consumers reported that meat could be aged and still considered “fresh”. The undesirability of frozen product poses a considerable barrier for export of meat from Australia to Asia, as short shelf life combined with long distances has made freezing a necessity.

It was also observed that both groups, when assessing sheepmeat, tended to extrapolate expectations of tenderness and flavour from previous experience with beef. For example, dry-aged sheepmeat is a novel product in the Australian market, and while neither group had experience with this product, both assessed the dry-aged sheepmeat image as premium.

### 3.3. Quantitative Result

#### 3.3.1. Demographics

For the entire consumer group (*n* = 75), the gender split was males 45%, females 55%; 0.8% of the group fell into the 18 to 19 year old category, 10% into the 20–25 year old category, 12% into the 26–30 year old category, 12% in the 31-39 year old category, 46% into the 40–60 year old category, and 11% in the 61–70 year old category. For the purposes of statistical analysis, the age categories were condensed into two categories of 18–30 and 31–70 years old which represented 29% and 71% of the consumers, respectively. Most consumers described their cultural heritage as Australian (70%), followed by British descent (12%), Asian descent (8%), European descent (4%), and Other (5%). The demographic distribution was similar between the beef and sheep tasting groups, and detailed demographic data can be found in Supplementary D.

#### 3.3.2. Eating Quality, Healthiness and Premiumness

When the data were analysed separately for each species for the effect of ageing method, the sheepmeat sensory data had no significant differences between the two samples (wet- and dry-aged; *p* > 0.05) for any of the eating quality parameters (tenderness, overall liking, flavour, juiciness, odour liking, SEQ), or for healthiness and premiumness (data not reported). For beef, the wet-aged samples were more tender than the dry-aged samples (*p* = 0.022, data not presented) with no other sensory traits being different between wet- and dry-aged beef (*p* > 0.05).

As no significant effects were found due to ageing method for sheepmeat and only for tenderness of beef, the data were further interrogated a priori for indications of consumer segmentation. Due to location challenges, an Asian cohort of appropriate size was not available for the sensory sessions, so analysis of cultural heritage effects was not possible. Other demographic effects were investigated and are described below.

For sheepmeat, it was found that females scored tenderness higher and tended to rate premiumness higher than males (*p* = 0.028 and *p* = 0.058 respectively, data not presented). Consumer age also affected consumer scores or sheepmeat for tenderness, juiciness, odour liking, SEQ, and healthiness, and tended to affect scores for overall liking, flavour, and premiumness, with older consumers (31–70 years) scoring these qualities higher than younger consumers (18–30 years), (Table 3). No significant age category effects were found for beef (*p* > 0.05), (Table 3).

#### 3.3.3. Quality Gradings, WTP (Willingness to Pay) and LTP (Likelihood to Purchase)

During the sensory sessions consumers were asked to select a quality grade (“unsatisfactory”, “good everyday quality”, ‘better than everyday quality” or “premium quality”) for each sample tasted. At the end of the sensory session, willingness and likelihood to purchase for each of the quality grades were indicated by all participants (these results are summarised in Table 4). Wet-aged beef was most likely to be rated as “better than everyday quality”, while the dry-aged beef was most likely to be rated as “good everyday quality”, and a similar pattern was observed for the sheepmeat samples. Consumers were willing to pay, on average, up to 50–60 AUD per kg for premium quality beef and 30–40 AUD per kg for premium quality sheepmeat, with prices decreasing with quality grade. Likelihood to purchase data indicated that consumers were less likely to purchase unsatisfactory meat even if it was priced considerably lower than higher quality meat, and this was especially evident for sheepmeat where mean consumer likelihood estimates for unsatisfactory meat were only 16% compared to 32% for beef.

**Figure 1:**
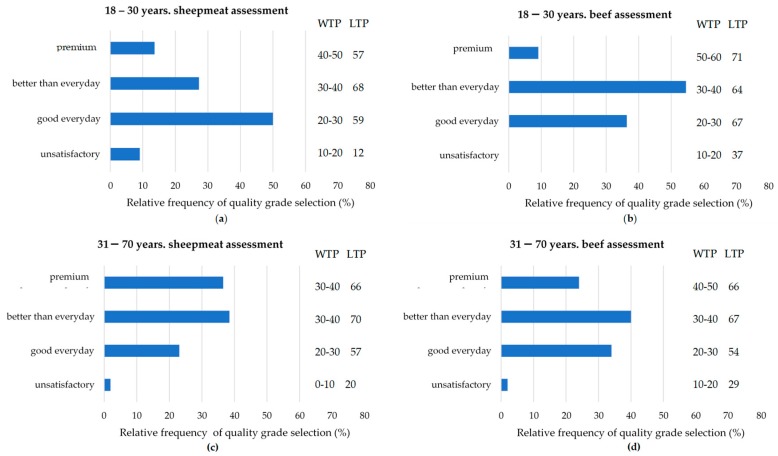
Relative frequency of quality grade selection (%, “premium”, better than everyday”, “good everyday”, “unsatisfactory”), willingness to pay (WTP; in AUD per kg.) and likelihood to purchase (LTP; %) for each quality grade split on age category and species. (**a**) 18–30 years, sheepmeat assessment; (**b**) 18–30 years, beef assessment; (**c**) 31–70 years, sheepmeat assessment; (**d**) 31–70 years, beef assessment.

The WTP and LTP consumer data for each of the quality grades were also segmented by age using the categories 18–30 and 31–70 years, and differences were found for the age groups (Figure 1). Older consumers were more likely to rate sheepmeat as a higher quality grade than younger consumers, with older consumers most frequently selecting “better than everyday quality”, and “premium quality”, while younger consumers most frequently selected “good everyday quality”. This effect was not found in the beef data. Older consumers (31–70 years) also indicated they would pay less than the younger consumers (18–30 years), for “premium quality” meat (30–40 AUD per kg vs. 40–50 AUD per kg, respectively, for sheepmeat and 40–50 AUD per kg vs. 50–60 AUD per kg, respectively, for beef) (Figure 1). The older consumers also differed from the younger consumers in WTP for “unsatisfactory” graded sheepmeat, with older consumers willing to pay ~10 AUD per kg less than younger consumers (0–10 AUD per kg vs. 10–20 AUD per kg, respectively). The age groups did not differ for WTP for “good everyday quality” and “better than everyday quality” grades for both sheepmeat and beef. Likelihood to pay results also indicated some differences between the two age groups, with younger consumers less likely to purchase “unsatisfactory” grade sheepmeat than older consumers (12% likelihood vs. 20% likelihood).

## 4. Discussion

Consumers are not a single homogenous group, and there is a substantial body of work demonstrating segmentation within consumer study populations. These segments exhibit differences in attitudinal and sensory responses to red meat [6,8,49,50,51,52,53,54,55,56,57]. Prescott and Bell [6] proposed that consumer preference is the result of food experience and this, in turn, is determined by cultural heritage. Indeed, Font-I-Furnols et al. [56] found that while Spanish consumers generally prefer the meat from lighter lambs, a small minority of Spanish consumers demonstrated a clear preference for meat from heavier lambs. German and British consumers were found to prefer meat from heavier lambs [53].

In this study, qualitative research revealed underlying differences in Asian and Australian consumer responses to red meat and these differences between cultures were similar for the two meat species investigated. For instance, Australian consumers’ preference for “Australian” labels was not reflected by their Asian counterparts, nor did Asians exhibit the Australians need to know the cut identity before they could assess eating quality.

The two groups also responded differently to unfamiliar cuts and concepts. Australians tended to move the unfamiliar into the everyday space, and in general were more skeptical of the unfamiliar, whereas the Asian group tended to map unfamiliar cuts and concepts into the premium space. This was especially apparent with the beef concepts; most were unfamiliar for the Asian group, but this presented no barrier to mapping them as premium. These results are in contradiction to previous views that unfamiliarity can be a barrier to consumer acceptance [28,30], thus highlighting the importance of investigations on preferences of specific groups of consumers and products. Ashman [58], based on the work of Von Hippel [59], reported that Australians are more conservative than Chinese consumers when considering new food products and estimated the ‘lead user’ population of Australia was only 12% compared to the Chinese ‘lead user’ population of 47%, which may explain some of the differences observed in this study. Furthermore, visual meat quality cues were found to be a potential barrier for Asian consumers. Pale colours were associated with frozen products, which had a lower acceptability. The Australian consumers in this study did not demonstrate the same sensitivity to colour.

While there were overlaps in what constitutes “premiumness” for the different cultures, there was evidence that the premium experience may be accessed via different channels with Asians going to the restaurant to experience premium meat as a professionally prepared hero ingredient. Although Australians also go to the restaurant, they are more likely to purchase expensive premium products for special occasions from the butcher, to cook at home. From a marketing perspective, the data suggest that both butchers and foodservice professionals play an important role in delivering a “premium“ meat experience/product for the Australian market, while foodservice facilities specialising in meat (as the hero ingredient) should be used to deliver the premium experience for Asian consumers.

Concept testing results indicated that when marketing products for Asian export markets, information indicating craftsmanship, rarity and personal quality (e.g., “small family farm produced”) provides a compelling story for these consumers and significantly enhances the perception of premium. 

Quantitative data indicated no significant differences in consumer eating quality, healthiness and premiumness scores for sheepmeat samples, and in the case of beef, only tenderness was different with the wet-aged sample (marble score = 500), preferred over the dry-aged sample (marble score = 300), likely because marbling increases meat tenderness and hence consumer tenderness scores [60].

The a priori segmentation analysis of the quantitative sheepmeat data indicated female consumers gave higher scores for sheepmeat tenderness and tended to score premiumness higher than male consumers. Older consumers (31–70 years) also scored sheepmeat higher than the younger consumers (18–30 years) for tenderness, juiciness, odour liking and health, while no effects were found for beef. There is evidence of differences in the drivers of liking according to meat species, with tenderness identified as the most important driver for beef, and flavour the most important driver for sheepmeat [61]. Sheepmeat has an intense flavour, and the aroma/flavour of sheepmeat can be a barrier to consumer acceptance, especially when unfamiliar, and thus consumers are not habituated [7,9]. It is proposed the sheepmeat assessments found in this study reflect the polarising nature of the consumers response to its aroma/flavour. Possibly, younger consumers were not as accustomed as the older consumers to sheepmeat. Watkins et al. [9] reported that consumers need to be habituated to eating sheepmeat in order to like it. This effect was also reflected in the quality grades assigned to sheepmeat by the two different age groups; older consumers (31–70 years) were more likely to rate sheepmeat as “better than everyday quality” and “premium quality”, while younger consumers (18–30 years) were more likely to rate it one grade lower, as “good everyday quality”.

Additionally, the a priori segmentation of WTP data indicates that the older consumer group was not prepared to pay as much as the younger consumer group for “premium quality” meat, 30–40 AUD per kg vs. 40–50 AUD per kg for sheepmeat, and 40–50 AUD per kg vs. 50–60 AUD per kg for beef. 

Similar gender and age effects to those found in this study have been reported previously. Kubberod et al. [55] demonstrated that young females preferred meat with a low intensity flavour and aroma compared to their male counterparts. Lyford et al. [8] reported that younger consumers (age range 25–35 years) in Australia, USA, Japan and Ireland were willing to pay more than older consumers for quality meat as did, Strydom et al. [62], in a study of South African consumers.

A final observation on the consumer sampling utilised for both the qualitative and quantitative panels was that it led to underrepresentation of some demographic groups. For example, as discussed above, the Asian cohort was not large enough to enable a cross-cultural comparison in the quantitative sensory sessions. Targeted consumer recruitment according to demographic profiles would enhance the mixed method approach described.

## 5. Conclusions

Coupling qualitative and quantitative methods provided an effective approach to investigate consumer preference for different meat products. This combined approach was able to differentiate consumer preferences from cultural and demographic perspectives while providing valuable insights into the underlying reasons for the differences. These insights can be used to inform product positioning strategies and to increase the competitiveness of Australian meats in both domestic and overseas markets.

Qualitative research demonstrated that factors influencing acceptability and rating of meat products differed between Australian and Asian consumers. These factors include colour, labelling information (including product quality, origin and craftmanship), domestic utility, bone and fat content, familiarity and access channel. Quantitative sensory analysis demonstrated that consumers were not able to differentiate the wet- and dry-aged sheepmeat samples for eating quality, premiumness or healthiness while the wet- and dry-aged beef samples were only differentiated for tenderness. A priori segmentation analysis of the demographic suggests gender and age may affect acceptability of sheepmeat among Australian consumers, and further study is warranted.

The mixed method approach described could be further improved by the use of stratified consumer populations that reflect target market demographics, especially as Asia encompasses a number of large markets with diverse religious backgrounds and cuisines. This study has highlighted the diversity of Australia’s local and export market consumers and the need for further targeted investigations on consumer response to Australian meat products.

## Figures and Tables

**Table 1:** Beef concepts, presentation order and their associated provenance attributes.

Order of Presentation	Concept	Provenance Factors
1	Premium pasture-fed beef from Blackmore’s Wagyu, Cape Grim or Minderoo	Established Premium Australian beef Brands
2	Fresh Australian Beef	Country of origin
3	Traditional Australian breeds like Brahman or Angus	An incongruent/unfamiliar breed claim, Angus and Braham breeds do not originate from Australia
4	Certified Organic Australian Beef	Generic Australian organic beef statement
5	Raised on a small family farm grass-fed using biodiverse pastures, hormone free and sustainable farming practices	Welfare and sustainability claims
6	Aged using traditional craftmanship practices like dry aging for 35 days to tenderise and create a distinctive melt in your mouth flavour	Artisan/craftmanship and eating quality claims
7	Unique breeds like older Longhorn that have a chance to develop more flavour, with a delicate beefy flavour and a slightly acid finish without having a very high fat content	Unique breed, sustainability, health and eating quality claims
8	Lean, heart-healthy beef, raised to have monosaturated fats to lower your blood pressure and cholesterol, but still have lots of flavour	Health and eating quality claim
9	Highest quality premium meat, recommended by celebrities, and chefs as their favourite	Generic claim of premium based on celebrity endorsement without any provenance information

**Table 2:** Top three product descriptors selected by the panel in order of preference, (selected from 28) descriptors for sheepmeat.

Order of Preference *	Australian Group 1	Australian Group 2	Asian Group 1	Asian Group 2
1	Australian	Australian	Fresh	Organic
2	Spring Lamb	Organic	Tender	Fresh
3	Tender/fresh	Tasty	Tasty	Aged

* 1 = most important, 2 = second most important, 3 = third most important.

**Table 3:** Effect of consumer age category (18–30 or 31–70 years) on consumer scores for eating quality (tenderness, overall liking, flavour, juiciness, odour liking and MQ4/SEQ), healthiness, and premiumness according to meat species (beef, sheep).

Attribute	Sheepmeat				Beef			
	18–30 years	31–70 years	*p*-Value	SED	18–30 years	31–70 years	*p*-Value	SED
Tenderness	66.7	77.9	0.042	5.28	77.5	70.7	0.202	5.28
Overall liking	69.2	78.0	0.053	4.40	75.8	71.8	0.334	4.17
Flavour	66.9	77.1	0.057	5.22	78.0	69.5	0.112	5.25
Juiciness	70.5	79.5	0.029	3.97	78.3	70.8	0.154	5.13
Odour liking	55.8	74.6	0.005	6.27	72.6	68.7	0.522	6.06
MQ4/SEQ *	67.9	77.9	0.026	4.28	77.2	70.8	0.156	4.43
Healthiness	60.1	75.9	0.004	5.10	75.1	705	0.339	4.69
Premiumness	68.8	75.8	0.081	3.89	75.8	69.6	0.227	5.00

Consumer scores ranged from 0–100; the higher the value, the more the attribute was appreciated. SED is the standard error of differences. * MQ4 = Meat quality combined score for beef. SEQ = Sheepmeat eating quality score. See Section 2.2.3 for calculation of both.

**Table 4:** Frequency of selecting a quality grade (“unsatisfactory”, “good everyday quality”, “better than everyday quality”, or “premium quality”) within each meat species (sheep or beef) according to ageing method (wet or dry) and associated willingness to pay data, indicated by median price category (0–10, 10–20, 20–20, 30–40, 40–50, 50–60 or 60–70, Australian dolllars (AUD) per kg) and the average likelihood of purchasing.

Meat Species	Quality Grade	Relative Frequency of Quality Grade Selection (%)		Median Price Category (AUD Per kg)	Average Likelihood of Purchasing
		Dry-aged	Wet-aged		(%)
Sheep	Unsatisfactory	0	3	0–10	16
	Good everyday quality	47	22	20–30	53
	Better than everyday quality	33	56	30–40	53
	Premium quality	19	19	30–40	58
Beef	Unsatisfactory	3	8	10–20	32
	Good everyday quality	42	21	20–30	58
	Better than everyday quality	25	44	30–40	66
	Premium quality	31	28	50–60	67

## Supplementary Materials

The following are available online at https://www.mdpi.com/2304-8158/9/2/126/s1, Supplementary A. Discussion guides, including Table SA1. Sheepmeat familiarity, Table SA2. Beef familiarity. Supplementary B. Stimulus design and source, including Table SB1. Stimulus descriptions and attributes design of experiment for sheepmeat, Table SB2. Sheepmeat descriptions and source, Table SB3. Stimulus descriptions and attribute design of experiment for beef, Table SB4. Beef stimulus descriptions and source. Supplementary C. Sheepmeat and beef perceptual mapping results, including Figure SC1. Exemplar maps from sheepmeat mapping exercise, Figure SC2. Exemplar map from group mapping beef exercise, Table SC1. Themes and group responses for sheepmeat perceptual mapping, Table SC2. Themes and group responses for beef perceptual mapping, Table SC3. Australian vs. Asian mapping of beef concepts in the quadrants of unfamiliar everyday, familiar everyday, unfamiliar premium, and familiar premium. Supplementary D. Demographic summary for sensory testing, including Table SD1. Number of participants per demographic category according to the meat species tested (sheepmeat or beef).
