# Supplementary material for: A Mixed Method Approach for the Investigation of Consumer Responses to Sheepmeat and Beef
Source: Foods. 2020 Jan 24;9(2):126. doi: 10.3390/foods9020126 (PMC7074361; doi:10.3390/foods9020126)
Supplement: Supplementary file 1 [file foods-09-00126-s001.docx]

**Supplementary A. Discussion guides**

# Table A1. Sheepmeat familiarity (Australia vs Asia)– Discussion Guide.

Objective: Determine **what cuts are most familiar,** and which cuts are considered the most premium? What variables matter? Fat content? Colour? Bone in bone out? Processing (frenched ?)

| **Stage** | **Time** | **Description** | **Objective** | **Key Questions** |
| --- | --- | --- | --- | --- |
| **Introduction** | **5min** | Housekeeping   - Introduce researchers - Confidentiality - Honesty – no right or wrong answers - Verbal Audio Recording & Photo permission | Meeting the respondent  & setting the scene |  |
| **Topic introduction**  **and concept intro** | **15mins** | How familiar are you with **sheep meat** products?  Review Concept:  Do you see lamb as a premium product?  Do you see mutton as a premium product?  What about  Hogget ? Goat? Spring lamb? | Understand where they currently sit in this space, history of usage | *Is* ***sheep meat*** *everyday (common) or premium?*  How often do you eat **sheep meat** in your household?  What is your favourite **sheep meat** dish?  What is your favourite **sheep meat** product?  What is the most expensive **sheep meat** product?   - How often would you buy it? - At what time of year/for what event?   Do you **sheep meat** in restaurants?  Have you ever had a bad **sheep meat** eating experience?   - What do you find most distinctive about eating **sheep meat?** |

# Table A1. Sheepmeat familiarity (Australia vs Asia)– Discussion Guide cont.

| **Stage** | **Time** | **Description** | **Objective** | **Key Questions** |
| --- | --- | --- | --- | --- |
| **Meat Attributes** | **45mins** | **Do not tell them what they are looking at.**  **Show them a range of …**  Primals and cuts  Processing  Colour and Fat  Glossiness  Browning  Images of meat, what works, does not work, when used, how to make them more ideal, best fit to concepts  Group Questions  Current products, most likely to buy? Least? assuming same cost (actual products) | Map set of primals and cuts against everyday to premium  familiar to unfamiliar  To understand reactions to key meat product attributes and fit to ideal | What makes a product familiar?  What makes a product premium?  Why is a product everyday?  What variables matter? Fat content? Colour? Bone in bone out? Processing (frenched ?) |

# Table A1. Sheepmeat familiarity (Australia vs Asia)– Discussion Guide cont.

| **Stage** | **Time** | **Description** | **Objective** | **Key Questions** |
| --- | --- | --- | --- | --- |
|  | **20mins** | **Word map**  Health and Wellness, Family, natural etc  What fits/ does not fit  Fit to target, make more ideal  Target explanation print out  Fit in Common, Premium, Health, Indulgent  **Especially interested in dry aged.**  **Lastly, lets map a picture again and call it spring lamb.**  **Let’s map the dry aged one again as well.** | Reactions to benefits and fit to ideal  Understand “the ideal“ for sheep meat  Are there perceived health benefits to sheep meat? |  |

# Table A2. Beef familiarity (Australia vs Asia )– Discussion Guide.

Objective: Determine **what cuts are most familiar,** and which cuts are considered the most premium? WHY- What variables matter? Fat content? Colour? Bone in bone out? Processing dry aged?

| **Stage** | **Time** | **Description** | **Objective** | **Key Questions** |
| --- | --- | --- | --- | --- |
| **Introduction** | **5min** | Housekeeping   - Introduce researchers - Confidentiality - Honesty – no right or wrong answers - Verbal Audio Recording & Photo permission | Meeting the respondent  & setting the scene |  |
| **Topic Introduction**  **and concept intro** | **15mins** | How familiar are you with **beef** products?  Review Concept/ context  Do you see beef as a premium product?  How does beef compare to lamb for premiumness?  Why? | Understand where they currently sit in this space, history of usage | *Is* ***beef*** *everyday (Common) or premium?*  How often do you eat **beef** in your household?  What is your favourite **beef** dish?  What is your favourite **beef** product?  What is the most expensive **beef** product?   - How often would you buy it? - At what time of year/for what event?   Do you **eat beef** in restaurants?  Have you ever had a bad **beef** eating experience?  What do you find most distinctive about eating **beef?** |

# Table A2. Beef familiarity (Australia vs Asia )– Discussion Guide cont.

| **Stage** | **Time** | **Description** | **Objective** | **Key Questions** |
| --- | --- | --- | --- | --- |
| **Meat Attributes** | **30mins** | **Do not tell them what they are looking at.**  **Show them range of …**  cuts  Colour and Fat  Browning  Frozen – what does fresh mean?  **Group Questions**  Current products, most likely to buy? Least? assuming same cost (actual products)  Some word slides can be used with the pictures 36, 37 38 | Map set of primals and cuts against everyday to premium  Familiar to unfamiliar  To understand reactions to key meat product attributes and fit to ideal | What makes a product familiar?  What makes a product premium?  Why is a product every day?  What variables matter? Fat content? Colour? bone in bone out  Dry aging? |
|  | **20mins** | **Word slides**  **Map and discuss why** | Map everyday to premium  Familiar to unfamiliar | *Why?* |

**Supplementary B. Stimulus design and source.**

**Table B1.** Stimulus descriptions and attribute design of experiment for sheepmeat.

| Sample Order | Sample cut / descriptor | Lean meat appearance | | | | | | Subcutaneous fat level | | | Bone | | Colour of fat | | | IIMF* | | Cut location | | | | | | | | |
| --- | --- | --- | --- | --- | --- | --- | --- | --- | --- | --- | --- | --- | --- | --- | --- | --- | --- | --- | --- | --- | --- | --- | --- | --- | --- | --- |
|  |  | dark | pale | grey/brown | brick red | glossy coating | not glossy | high | medium | low | yes | no | white | pink | yellow | low | high | leg | loin | shank | breast | neck | tenderloin | rack | shoulder | sirloin |
| 1 | Non-glossy broken leg |  |  |  |  |  | X |  | X |  | X |  |  |  |  | X |  | X |  |  |  |  |  |  |  |  |
| 2 | Diced lamb |  |  |  | X | X |  |  |  | X |  | X | X |  |  | X |  |  | N |  |  |  |  |  |  |  |
| 3 | Whole loin | X |  |  |  |  | X |  |  | X | X |  |  |  | X | X |  |  | X |  |  |  |  |  |  |  |
| 4 | Lamb mince |  |  |  | X |  | X |  |  |  |  | X | X |  |  |  |  |  | N |  |  |  |  |  |  |  |
| 5 | Shanks | X |  |  |  |  | X |  |  | X | X |  | X |  |  |  |  | X |  |  |  |  |  |  |  |  |
| 6 | Whole shoulder |  |  |  | X |  | X |  |  |  | X |  |  |  | X |  |  |  |  |  |  |  |  |  | X |  |
| 7 | Sirloin | X |  |  |  |  | X |  |  | X |  |  | X |  |  | X |  |  |  |  |  |  |  |  |  | X |
| 8 | Tenderloin | X |  |  |  |  | X | X |  |  |  | X | N |  |  | X |  |  |  |  |  |  |  |  |  |  |
| 9 | Leg steak | X |  |  |  |  | X |  |  | X | X |  | X |  |  | X |  | X |  |  |  |  |  |  |  |  |
| 10 | Neck chop |  |  |  | X |  | X | X |  |  | X |  | X |  |  | X |  |  |  |  |  | X |  |  |  |  |
| 11 | Boneless leg rolled with string | X |  |  |  |  | X |  | X |  |  | X |  | X |  | X |  | X |  |  |  |  |  |  |  |  |
| 12 | Short ribs |  |  |  | X |  | X | X |  |  | X |  | X |  |  |  |  |  |  |  | X |  |  |  |  |  |
| 13 | Shoulder rack (frenched) |  | X |  |  |  | X |  |  | X | X |  | X |  |  | X |  |  |  |  |  |  |  |  | X |  |
| 14 | Chinese rolled leg meat | X |  |  |  |  | X | X |  |  |  | X | X |  |  | X |  | X |  |  |  |  |  |  |  |  |
| 15 | Lamb strips | X |  |  |  | X |  |  |  | X |  | X | N |  |  | X |  |  | N |  |  |  |  |  |  |  |
| 16 | Mutton blocks frozen | X |  |  |  |  | X | X |  |  |  | X | X |  |  |  | X | X |  |  |  |  |  |  |  |  |
| 17 | Chinese whole ribs | X |  |  |  |  | X | X |  |  | X |  | X |  |  |  | X |  | X |  |  |  |  |  |  |  |

**Table B1.** Stimulus descriptions and attribute design of experiment for sheepmeat cont.

| Sample Order | | Sample cut / descriptor | Lean meat appearance | | | | | | Subcutaneous fat level | | | Bone | | Colour of fat | | | IIMF* | | Cut location | | | | | | | | |
| --- | --- | --- | --- | --- | --- | --- | --- | --- | --- | --- | --- | --- | --- | --- | --- | --- | --- | --- | --- | --- | --- | --- | --- | --- | --- | --- | --- |
|  |  |  | dark | pale | grey/brown | brick red | glossy coating | not glossy | high | medium | low | yes | no | white | pink | yellow | low | high | leg | loin | shank | breast | neck | tenderloin | rack | shoulder | sirloin |
| 18 | Deboned leg loose | |  | X |  |  |  | X |  |  | X |  | X | X |  |  | X |  | X |  |  |  |  |  |  |  |  |
| 19 | 3 T-bone chops | | X |  |  |  |  | X |  | X |  | X |  | X |  |  | X |  |  | X |  |  |  |  |  |  |  |
| 20 | Frenched rack high IMF* | |  |  |  | X |  | X |  | X |  | X |  | X |  |  |  | X |  |  |  |  |  |  | X |  |  |
| 21 | Frenched rack low IMF* | |  |  |  | X | X |  |  |  | X | X |  | X |  |  | X |  |  |  |  |  |  |  | X |  |  |
| 22 | Forequarter chop, not fresh | |  |  | X |  | X |  |  | X |  | X |  |  | X |  |  | X |  |  |  |  |  |  |  | X |  |
| 23 | Forequarter chop, odd shape, not fresh | |  |  | X |  | X |  |  | X |  | X |  |  | X |  |  | X |  |  |  |  |  |  |  | X |  |
| 24 | Dry aged mutton | | X |  | X |  |  | X | X |  |  |  | X | X |  |  |  | X |  |  |  |  |  |  |  |  | X |
| 25 | Medallions | |  |  | X | X | X |  |  |  | X |  | X | X |  |  | X |  |  |  |  |  |  | X |  |  |  |

N = attribute not relevant, IMF* =Intramuscular fat

**Table B2.** Sheepmeat stimulus descriptions and source.

| **Sample Order** | **Sample cut / descriptor** | **Image source** |
| --- | --- | --- |
| 1 | Non-glossy broken leg | Authors image collection |
| 2 | Diced lamb | <https://glennlewinbutchers.co.uk/collections/lamb/products/diced-lamb-500g> |
| 3 | Whole loin | <http://www.foodsubs.com/MeatLambLoin.html> |
| 4 | Lamb mince | <http://www.scibbosqualitymeats.com.au/store/lamb-mince/> |
| 5 | Shanks | https://richmeats.capetown/wp-content/uploads/2017/04/199-1-1024x683.jpg |
| 6 | Whole shoulder | http://www.foodsubs.com/Photos/lambshoulder.jpg |
| 7 | Sirloin | http://www.foodsubs.com/Photos/lambsirloinroast.jpg |
| 8 | Tenderloin | http://www.mulwarra.com.au/wcontent/uploads/2015/09/5082-Lamb-Tenderloin-Butt-Off.jpg |
| 9 | Leg steak | http://files.recipetips.com/images/glossary/l/lamb_leground.jpg |
| 10 | Neck chop | https://dtgxwmigmg3gc.cloudfront.net/imagery/assets/derivations/icon/256/256/true/eyJpZCI6IjNmNWU2NThmZmQwMzg4NWExMTM0OTU3OWNiM2E0MTE5Iiwic3RvcmFnZSI6InB1YmxpY19zdG9yZSJ9?signature=46e41a8f52f042b685f028b71e1280a83ec5033fd5abe47affa91d9b9ae2ab41 |
| 11 | Boneless leg rolled with string | <https://www.abelandcole.co.uk/organic-half-leg-of-lamb-boneless-800g> |
| 12 | Short ribs | http://trolley.ae/image/data/butchery/113974.jpg |
| 13 | Shoulder rack (frenched) | http://www.mulwarra.com.au/wp-content/uploads/2015/09/4739-Lamb-Shoulder-Rack-Frenched.jpg |
| 14 | Chinese rolled leg meat | http://gaitaobao2.alicdn.com/tfscom/i4/TB1b1.1JXXXXXXgXpXXXXXXXXXX_%21%210-item_pic.jpg_310x310.jpg |
| 15 | Lamb strips | https://meatperfection.com.au/store/lamb-strips |
| 16 | Mutton blocks frozen | http://gaitaobao2.alicdn.com/tfscom/i1/TB19Zu7KFXXXXaqXFXXXXXXXXXX_%21%210-item_pic.jpg_310x310.jpg |
| 17 | Chinese whole ribs | http://gaitaobao2.alicdn.com/tfscom/i4/TB1PUBINVXXXXXWXXXXXXXXXXXX_%21%210-item_pic.jpg_310x310.jpg |
| 18 | Deboned leg loose | https://img.alicdn.com/bao/uploaded/i4/TB15kK1NXXXXXXGapXXXXXXXXXX_%21%210-item_pic.jpg_310x310.jpg |
|  |  |  |
|  |  |  |
|  |  |  |
|  |  |  |

**Table B2.** Sheepmeat stimulus descriptions and source cont.

| **Sample Order** | **Sample cut / descriptor** | **Image source** |
| --- | --- | --- |
| 19 | 3 T-bone chops | http://www.greatbritishhalalmeat.co.uk/wp-content/uploads/2014/12/lambchops.jpg |
| 20 | frenched rack high IMF* | https://img1.wsimg.com/isteam/ip/beb56668-d24f-4d6f-87dd-47dee557bf50/7afc3574-d62b-46e3-8fcb-3c4c23748d45.jpg/:/cr=t:2.83%25%2Cl:0%25%2Cw:100%25%2Ch:94.34%25/rs=w:458%2Ch:229%2Ccg:true |
| 21 | frenched rack low IMF* | https://fthmb.tqn.com/RQgvwjEn5uQEVWCyN92otFJLGhY=/350x0/filters:no_upscale()/rack-of-lamb-56a5b5445f9b58b7d0de0d84.jpg |
| 22 | forequarter chop, not fresh | Author’s image collection |
| 23 | forequarter chop, odd shape, not fresh | Author’s image collection |
| 24 | dry aged mutton | Author’s image collection |
| 25 | medallions | http://www.meatcart.com.au/media/catalog/product/cache/1/small_image/218x/9df78eab33525d08d6e5fb8d27136e95/l/a/lamb-medallion-small.jpg |

**Table B3.** Stimulus descriptions and attribute design of experiment for beef.

| Sample Order | Sample cut / descriptor | Lean meat appearance | | | | | | Subcutaneous fat level | | | Bone | | Colour of fat | | | IIMF* | | Cut location | | | | | | |
| --- | --- | --- | --- | --- | --- | --- | --- | --- | --- | --- | --- | --- | --- | --- | --- | --- | --- | --- | --- | --- | --- | --- | --- | --- |
|  |  | dark | pale | grey/brown | brick red | glossy coating | not glossy | high | medium | low | yes | no | white | pink | yellow | low | high | leg | loin | shank | brisket | neck | Cuck | Sirloin |
| 0 | Porterhouse steak (image ref 9 on map) |  | X |  |  | X |  | X |  |  |  | X | X |  |  | X |  |  | X |  |  |  |  |  |
| 1 | Beef neck |  |  | X |  |  | X |  | X |  | X |  |  | X |  | X |  |  |  |  |  | X |  |  |
| 2 | Oxtail |  |  |  | X |  | X | X |  |  | X |  | X |  |  | X |  |  |  |  |  |  |  |  |
| 3 | T-bone steak | X |  |  |  |  | X |  |  | X | X |  |  | X |  |  | X |  | X |  |  |  |  |  |
| 4 | Scotch fillet |  |  |  | X |  | X |  |  | X |  | X | X |  |  |  | X |  | X |  |  |  |  |  |
| 5 | Whole eye fillet |  | X |  |  |  | X |  |  | X |  | X | X |  |  |  | X |  | X |  |  |  |  |  |
| 6 | Dry aged rib eye | X |  |  |  |  | X |  |  |  | X |  |  |  | X |  |  |  | X |  |  |  |  |  |
| 7 | Striploin | X |  |  |  |  | X |  | X |  |  | X | X |  |  | X |  |  | X |  |  |  |  |  |
| 8 | Cube roll |  |  |  | X |  | X | X |  |  |  | X | N |  |  | X |  |  | X |  |  |  |  |  |
| 10 | Wagyu striploin | X |  |  |  |  | X | X |  |  |  | X | X |  |  |  | X |  | X |  |  |  |  |  |
| 11 | Round steak |  |  |  | X |  | X |  | X |  |  | X |  |  | X | X |  | X |  |  |  |  |  |  |
| 12 | Osso Bucco |  | X |  |  | X |  |  |  | X | X |  | X |  |  |  |  | X |  |  |  |  |  |  |
| 13 | Flower marbled sirloin from Hanwoo | X |  |  |  |  | X |  |  | X |  | X | X |  |  |  | X |  |  |  |  |  |  | X |
| 14 | Beef cheek |  |  | X |  |  | X |  |  | X |  | X | X |  |  | X |  | N |  |  |  |  |  |  |
| 15 | Diced beef | X |  |  |  | X |  |  |  | X |  | X | N |  |  | X |  |  | N |  |  |  |  |  |
| 16 | Beef back ribs | X |  |  |  | X |  |  |  | X | X |  | X |  |  |  | X |  | X |  |  |  |  |  |
| 17 | Discoloured rib eye due to MAP**  atmosphere packaged beef |  |  | X |  |  | X | X |  |  |  | X | X |  |  |  | X |  | X |  |  |  |  |  |

**Table B3.** Stimulus descriptions and attribute design of experiment for beef cont.

| Sample Order | Sample cut / descriptor | Lean meat appearance | | | | | | Subcutaneous fat level | | | Bone | | Colour of fat | | | IIMF* | | Cut location | | | | | | |
| --- | --- | --- | --- | --- | --- | --- | --- | --- | --- | --- | --- | --- | --- | --- | --- | --- | --- | --- | --- | --- | --- | --- | --- | --- |
|  |  | dark | pale | grey/brown | brick red | glossy coating | not glossy | high | medium | low | yes | no | white | pink | yellow | low | high | leg | loin | shank | brisket | neck | Cuck | Sirloin |
| 18 | Beef mince |  |  |  | X |  | X |  | X |  |  | X | X |  |  | N |  | X |  |  |  |  |  |  |
| 19 | Stringed cube roll roast |  |  |  | X |  | X |  | X |  |  | X | X |  |  | X |  |  | X |  |  |  |  |  |
| 20 | Fresh MAP** striploin |  |  |  | X |  | X |  |  | X |  | X | X |  |  | X |  |  | X |  |  |  |  |  |
| 21 | Aged MAP** striploin | X |  |  |  |  | X |  |  | X |  | X | X |  |  | X |  |  | X |  |  |  |  |  |
| 22 | Over aged MAP** striploin |  |  | X |  |  | X |  |  | X |  | X | X |  |  | X |  |  | X |  |  |  |  |  |
| 23 | Shaved beef slices |  | X |  |  |  | X | X |  |  |  | X |  | X |  |  | X | X |  |  |  |  |  |  |
| 24 | Beef strips | X |  |  |  |  | X |  |  | X |  | X | N |  |  | X |  | N |  |  |  |  |  |  |
| 25 | Frozen beef dice |  | X |  |  |  | X |  |  | X |  | X | N |  |  | X | X |  |  |  |  |  | X |  |

N = attribute not relevant, IMF* = Intramuscular fat, MAP** = Modified atmosphere packaging

**Table B4.** Beef stimulus descriptions and source.

| Sample Order    **Sample Order** | **Sample cut / descriptor** | **Image source** |
| --- | --- | --- |
| 0 | Porterhouse steak  (image ref 9 on map) | https://www.google.com.au/search?biw=1777&bih=852&tbm=isch&sa=1&ei=rCtQW63QKs3-0gTCrKroDQ&q=image+of+porterhouse+steak+raw&oq=image+of+porterhouse+steak+raw&gs_l=img.3...15225.16595.0.17128.4.4.0.0.0.0.202.745.0j3j1.4.0....0...1c.1.64.img..0.1.202...0i30k1.0.aVEvuqq-Mx4#imgrc=2TgdpZzphTXcQM: |
| 1 | Beef neck | https://www.seriouseats.com/2011/08/the-nasty-bits-beef-neck-tacos.html |
| 2 | Oxtail | https://upload.wikimedia.org/wikipedia/commons/9/94/Raw_oxtail-01.jpg |
| 3 | T-bone steak | https://www.google.com.au/search?q=image+of+t+bone+steak&tbm=isch&source=iu&ictx=1&fir=oH3MpdrNZvjR7M%253A%252CqIECNKawwsPPxM%252C_&usg=__Mb5j0PR2finU-tH7_AtZkl2P-xs%3D&sa=X&ved=0ahUKEwj5qbDlvKrcAhVUCqYKHVs6D9wQ9QEILzAD#imgrc=oH3MpdrNZvjR7M:  T-Bone |
| 4 | Scotch fillet | https://www.google.com.au/search?q=image+of+rib+eye+steak&tbm=isch&tbo=u&source=univ&sa=X&ved=0ahUKEwim_pmEvarcAhUMj5QKHZOPD5gQsAQIJg&biw=1777&bih=852#imgrc=djqnJxTBd3-zoM: |
| 5 | Whole eye fillet | https://organicmeatonline.com.au/products/cape-grim-eye-fillet-grass-fed  Eye fillet |
| 6 | Dry aged rib eye | https://www.google.com.au/search?q=image+of+dry+aged+beef+rib+eye&tbm=isch&tbo=u&source=univ&sa=X&ved=0ahUKEwjPk9Kz4azcAhXLI5QKHR0TAcUQsAQIKA&biw=1206&bih=683#imgrc=NWsFpMYlSU2-pM: |
| 7 | Striploin | https://www.google.com.au/search?biw=1777&bih=852&tbm=isch&sa=1&ei=_CdQW6VvhezSBIOtsrgC&q=image+of+beef+striploin&oq=image+of+beef+striploin&gs_l=img.3...15144.20093.0.20629.14.14.0.0.0.0.327.2666.0j11j2j1.14.0....0...1c.1.64.img..0.5.1116...0j0i30k1j0i8i30k1j0i24k1.0.ZZ-7yfKvSUU#imgrc=8tzJ0rnUwLpDYM: |
| 8 | Cube roll | <https://www.supplybunny.com/en/products?category=beef-cube-roll> |
|  |  |  |

**Table B4.** Beef stimulus descriptions and source cont.

| **Sample Order** | **Sample cut / descriptor** | **Image source** |
| --- | --- | --- |
| 10 | Wagyu striploin | https://www.google.com.au/search?q=image+of+highly+marbled+striploin&tbm=isch&tbo=u&source=univ&sa=X&ved=0ahUKEwi_9ZTu3azcAhXFsJQKHemCBLAQsAQIKQ&biw=1206&bih=560&dpr=0.9#imgrc=vLCWyvpddSOkBM: |
| 11 | Round steak | https://organicmeat.ie/shop/shop/organic-beef/organic-round-steak |
| 12 | Osso Bucco | <http://meatperfection.com.au/store/beef-osso-bucco> |
| 13 | Flower marbled sirloin from Hanwoo | https://www.koreanbbqshop.com/kkotdeungsim/ |
| 14 | Beef cheek | https://www.google.com.au/search?q=image+of+beef+cheek&tbm=isch&source=iu&ictx=1&fir=mBntJRQMrbzNjM%253A%252CsQL49zf-IAxfBM%252C_&usg=__2C7bDMHAo_n58pEdlqQePWztKqE%3D&sa=X&ved=0ahUKEwjD3obErrTcAhVGx7wKHV73CrAQ9QEIOzAJ#imgrc=57hOhHM-wqoZqM: |
| 15 | Diced beef | https://www.google.com.au/search?q=image+of+diced+beef&tbm=isch&tbo=u&source=univ&sa=X&ved=0ahUKEwj75u_RvLTcAhWJv7wKHdxXAFcQsAQIJg&biw=1777&bih=852#imgrc=baunhk5vi5gO2M: |
| 16 | Beef back ribs | http://kamadojim.com/smoked-beef-back-ribs-recipe/ |
| 17 | Discoloured rib eye due to MAP**  atmosphere packaged beef | <https://www.seriouseats.com/2013/01/the-food-lab-dry-age-beef-at-home.html> |
| 18 | Beef mince | https://www.google.com.au/search?q=beef+mince+image&tbm=isch&tbo=u&source=univ&sa=X&ved=0ahUKEwig-bWBwLTcAhUL87wKHaJGCGoQsAQIJg&biw=1777&bih=852#imgrc=lvExfZBxrTabyM: |
| 19 | Stringed cube roll roast | <https://mansfieldiga.com.au/lines/roast-beef> |
| 20 | Fresh MAP** striploin | http://www.thebeefsite.com/articles/3295/predicting-tenderness-and-lasting-colour-in-meat/ |
| 21 | Aged MAP** striploin | http://www.thebeefsite.com/articles/3295/predicting-tenderness-and-lasting-colour-in-meat/ |
| 22 | Over aged MAP** striploin | http://www.thebeefsite.com/articles/3295/predicting-tenderness-and-lasting-colour-in-meat/ |
| 23 | Shaved beef slices | https://www.google.com.au/search?q=image+of+hotpot+beef&tbm=isch&tbo=u&source=univ&sa=X&ved=0ahUKEwjm6PabwrTcAh  VKxLwKHVM9C6gQsAQIKA&biw=1777&bih=852#imgrc=kk5C4zwI9T4YmM: |
| 24 | Beef strips | <https://organicmeatonline.com.au/collections/black-angus/products/grass-fed-organic-black-angus-thick-beef-stir-fry> |
| 25 | Frozen beef dice | <http://kenh14.vn/chan-ngay-voi-do-dong-lanh-roi-cho-xin-mot-ve-ve-noi-tuoi-ngot-thoi-20180530023715096.chn> |

**Supplementary C. Sheepmeat and beef perceptual mapping results**

**Table C1**. Themes and group responses for sheepmeat perceptual mapping.

| **Australian consumers** | **Asian consumers** |
| --- | --- |
| **Theme- Usage and Eating Occasions**  Lamb is eaten at home and at restaurants and purchased at supermarkets and butchers. Typically, Australians go to the butcher when looking to purchase special meat or for an occasion. They would also prefer to get diced meat prepared at the butcher. When purchasing meat, they use the label in the supermarket to determine the appropriate cooking method, or if purchasing from a butcher, they ask for cooking method recommendations. Cooking methods included roasting, slow cooking, grilling and BBQ. Large joints of meat are more special and prepared on the weekend when there is more time for preparation. | The Asian groups contained participants with diverse backgrounds including Indian, Chinese and ASEAN countries. Those from the Indian subcontinent were very familiar with eating sheep-meat both at home and out in restaurants (often in curry). For this group, the term “mutton” meant the meat from a goat. Participants from China and South East Asia did not eat sheepmeat regularly and most often in restaurants. Cooking method selection was a more straightforward decision process for this group with most in-home cooking done on the stove top rather than in an oven. |
| **Theme – Cuts**  These participants needed a label to identify the product so they could then decide how they would prepare it (e.g. roasting vs slow cooking vs grilling). Often they could not recognise the cut. The cooking method applied to the product was key for them to make the link from visual attributes to eating quality or eating occasion for the product. Loin cuts (the most expensive cuts in Australia) were the most familiar and premium. | Cuts were not as important for this group- choice was based on the dish they were going to prepare, and cooking method was linked to the dish, e.g. slice finely for frying or cut into cubes for slow cooking. Bone could be a bonus if they were going to make soup. Rack and cutlets were most premium (rack was very familiar, but cutlets were not.) |
| **Theme- Labelling**  The lack of labels was a significant barrier when attempting to map the products. The inability to identify the products and therefore how they would cook it complicated mapping (occasionally there were mis-identifications and incorrect assumptions made on how they would prepare the cut). | Asian participants were generally much more comfortable with assessing meat quality based on visual attributes than Australians – the lack of labelling did not hamper mapping, and they were quick to decide how they would cook meat based on how it looked. A range of uses for each cut was discussed during the mapping exercise, indicating they would choose the type of eating experience they wanted at the time of meal preparation |
| **Theme-Fat**  Fat content mattered to this group, most expressing a preference for leaner meat for health reasons and one participant describing themselves as fat phobic. The mutton roll was too fatty for this group: they would neither buy nor eat it. Upon presentation of the lean rack and the rack with high levels of intramuscular fat (IMF), the lean rack was more desirable to the group as it had a small amount of subcutaneous fat that would make the meat juicy upon roasting (as was the case with the rolled roast). The high IMF rack was less desirable for this group, and when questioned, they felt the eating of this rack would be less pleasurable as the marbling of fat could indicate stringiness. | As with the Australian group, too much visible fat made the product less premium, but a distinction was made between subcutaneous fat and IMF. Subcutaneous fat was undesirable for the Asian groups, but IMF was associated with improved eating quality. There were some inferences being made about premium beef with high IMF being very desirable; therefore, high IMF sheepmeat may be similarly desirable. |

**Table C1**. Themes and group responses for sheepmeat perceptual mapping cont.

| **Australian consumers** | **Asian consumers** |
| --- | --- |
| **Theme-Trimming**  Trimming makes a product more premium – French trim racks were the most premium cuts. Conversely, the whole loin that had not been trimmed was less premium than trimmed loin. Stringing also made the product more premium, because someone had made the effort to string it together and make it more presentable. | As with the Australian group, trimming made the racks more premium; however, the high IMF racks were most premium due to their superior eating quality |
| **Theme-Colour**  Response to colour was not as pronounced with this group as it was for the Asian groups. The not-so- fresh chops were detected by the western group as not fresh, and the soft pink colour of the de-boned leg was taken as an indicator of meat softness. The darkened colour of dry aged mutton was recognised as premium even though it was unfamiliar. | Colour was a key indicator for meat quality; dark brick red was fresher and more premium while pale flesh indicated the product might have been frozen and therefore was not fresh. The slightly spoiled chops were quickly identified as not fresh by the group and mapped with much less discussion compared to the non-Asian group. The darkened colour of dry aged mutton was recognised as premium even though it was unfamiliar. |
| **Theme-Tenderness**  Tenderness mattered to this group – there was a discussion on how tenderloin /eye fillet is more tender, and several participants were actively searching for these muscles in the range of cuts presented-this was based on their understanding of tenderness in beef. | Tenderness was not raised by this group- cooking time was extended if meat was not tender enough |
| **Theme-Value for money**  The “value for money” of the spare ribs was challenged because of the high proportion of bone to meat. Likewise, they questioned the value of paying for fat: flesh is the best part of the product. | Too much fat was a reason to pay less for meat as they would have to trim it off; however, the bone was not such an issue as it could be used in soups to add flavour. |
| **Theme-Convenience**  Convenient (quick to cook) meat is needed for the working week, so they would go for a boneless product or one that comes in small portion sizes. | The Asian consumers preferred deboned cuts for cooking at home, but they were comfortable slicing to the right size to cook. |
| **Theme-Dry aged**  Both groups recognised the darker colour of the dry aged mutton image as a premium product even though neither group has experience of this product | See Australian note |

Australian consumers

(**a**)

Asian consumers

(**b**)

**Figure C1**. Exemplar maps from sheepmeat mapping excericise. **a**. Australian group and **b**. Asian group. 1 = Broken leg (non glossy), 2 = Diced lamb, 3 = Whole loin, 4 = Lamb mince, 5 = Shanks, 6 = Whole shoulder, 7 = Sirloin, 8 = Tenderloin, 9 = Leg steak, 10 = Neck chop, 8 = Boneless leg rolled with string, 9 = short ribs, 10 = Sshoulder rack (frenched), 11 = Chinese rolled leg meat, 12 = Lamb strips, 13 = Chinese mutton blocks frozen, 14 = Chinese whole ribs, 15 = Deboned leg loose, 16 = T-bone chops, 17 = Frenched rack high intramuscular fat, 18 = Frenched rack low intramuscular fat, 19 = Forequarter chop, not fresh, 20 = Forequarter chop, odd shape, not fresh, 21 = Dry aged mutton, 22 = Medallions

**Table C2**. Themes and group responses for beef perceptual mapping.

| **Australian consumers** | **Asian consumers** |
| --- | --- |
| **Theme-Usage and eating occasions**  Most grew up eating meat and veg “the Aussie way” although most now cook cheaper meat at home like mince and have the more expensive cuts in restaurants because they can be tricky to cook. They all eat less of it then they used to. Family occasions tend to be roast lamb rather than roast beef. | “Cut selection is driven by the dish I am cooking”. “If it’s not familiar, I will eat it in the restaurant.” Large cuts are typically restaurant options.  “Irregular shapes make it suitable for BBQ-if I’m uncertain about the cut it’s good for BBQ.” |
| **Theme- Cuts**  Need to know the name of a cut so they can map it; there are expectations of eating quality associated with the cut name. Some cuts are avoided because they are hard to prepare at home, e.g. steak. “The more prep I do, the more special it is”, e.g. osso bucco, ribs. If it is not familiar, it is likely to go into everyday. If it has a fair proportion of bone, it is likely to go into everyday. | “Cut determines if I cook at home or go to the restaurant”.  “Selection of cut is based on the dish I am cooking, if it is not familiar I prefer in a restaurant”. From the map, if it is unfamiliar, it is likely to be mapped as premium. Premium cuts tend to be small portions high in intramuscular fat, of deeper red colour and suitable for a quick cook, fry or grill. |
| **Theme-Labelling**  Need to know the name of the cut so they can decide on mapping-some cuts are avoided as they can be hard to cook at home. In general, they were more familiar with beef cuts than sheep cuts. | Asian group mapped meat quickly and as with sheepmeat were comfortable assessing eating quality visually. All cuts with higher IMF made it to the premium side of the map. |
| **Theme-Fat**  The group is split on how it responds to fat:  1. Marbled fat means a premium taste  2. Visible fat means it is bad for you. If it’s subcutaneous fat, they will cut it off before cooking. | Marbled fat means a premium taste. Fat on the outer edge needs to be minimised. |
| **Theme-Trimming**  Australian consumers generally felt cuts with bone in are not good value; the exception was osso bucco which takes a lot of time to prepare and is special. | Cuts mapped as premium were all sliced into steak format or thinner (Waygu hot pot) small portions and were for grilling. The everyday side of the map included roasting joints, mine, dice and bone in cuts |
| **Theme-Colour**  The group is split on response to the dry aged beef image:   1. Dark colour- been in the fridge too long, not acceptable 2. Dark colour- could be aged but should be a more consistent dark colour   Grey colour is suspicious. | Red is fresh. Deep Red is organic. Pink is pork. Brown is unacceptable. |
| **Theme-Tenderness**  Most understood which cuts are more tender and these cuts made it to the premium side of the map. One participant admitted he preferred rump for flavour over the more tender cuts but understood he would be in the minority | Tenderness was not raised by this group. |
| **Theme-Value for money**  Bones make up the weight, and you don’t want to be paying for bone. | Too much fat was a reason to pay less for meat as they would have to trim it off; however, bone was not such an issue as it could be used in soups to add flavour. |

**Table C2**. Themes and group responses for beef perceptual mapping cont.

| **Australian consumers** | **Asian consumers** |
| --- | --- |
| **Theme-Convenience**  Roasting is too time consuming, and several would never buy a roasting joint. While they buy mince and dice for the convenience, they expect it to be low quality meat. | Asians preferred steak cuts for cooking at home, slicing to the right size to cook. Bigger deboned joints were for making curry. |
| **Theme-Dry aged**  The Australian group was skeptical of the colour; some recognised it as a premium product while others felt something had gone wrong somewhere with the meat storage. | Asian group recognised dry aged as premium but also unfamilar. |

Australian consumers

(**a**)

Asian consumers

(**b**)

**Figure C2;** Exemplar map from group mapping beef exercise**. a**. Australian group and **b**. Asian group.

1 = Beef neck, 2 = Oxtail, 3 = T-bone steak, 4 = Scotch fillet, 5 = Whole eye fillet, 6 = Dry aged rib eye, 7 = Striploin, 8 = Cube roll, 9 = Porterhouse steak, 10 = Wagyu striploin, 11 = Round steak, 12 = Osso Bucco, 13 = Flower marbled sirloin from Hanwoo, 14 = Beef cheek, 15 = Diced beef, 16 = Beef back ribs, 17 = Discoloured rib eye due to modified atmosphere packaging, 18 = Beef mince, 19 = Stringed cube roll roast, 20 = Fresh modified atmosphere packaged striploin, 21 = Aged modified atmosphere packaged striploin, 21 =Over aged modified atmosphere packaged striploin, 22 = Shaved beef slices, 23 = Beef strips, 24 = Frozen beef dice.

**Table C3.** Australian vs Asian mapping of beef concepts in the quadrants of unfamilar everyday, familiar everyday, unfamilar premium, and familiar premium.

| **Australian** | **Asian** |
| --- | --- |
| **Quadrant; Unfamiliar-Everyday**  Traditional Australian breeds like Brahman or Angus. | No phrase mapped in this space |
| **Quadrant; Familiar--Everyday**  Fresh Australian Beef | Fresh Australian Beef |
| **Quadrabt; Unfamilar-Premium**  Aged using traditional craftmanship practices like dry aging for 35 days to tenderise and create a distinctive melt in your mouth flavour | Aged using traditional craftmanship practices like dry aging for 35 days to tenderise and create a distinctive melt in your mouth flavour  Traditional Australian breeds like Brahman or Angus.  Raised on a small family farm grass fed using biodiverse pastures, hormone free and sustainable farming practices  Lean heart healthy beef, raised to have monosaturated fats to lower your blood pressure and cholesterol, but still have lots of flavour.  Certified Organic Australian Beef.  Unique breeds like older Longhorn that have a chance to develop more flavour, with a delicate beefy flavour and a slightly acid finish without having a very high fat content.  Highest quality premium meat, recommended by celebrities and chefs as their favourite.  Premium pasture fed beef from Blackmore’s Wagyu, Cape Grim or Minderoo |
| **Quadrant; Familiar-Premium,**  Certified Organic Australian Beef.  Unique breeds like older Longhorn that have a chance to develop more flavour, with a delicate beefy flavour and a slightly acid finish without having a very high fat content  Premium pasture fed beef from Blackmore’s Wagyu, Cape Grim or Minderoo  Raised on a small family farm grass fed using biodiverse pastures, hormone free and sustainable farming practices | No phrase mapped in this space |

**Supplementary D. Demographic summary for sensory testing**

**Table D1.** Number of participants per demographic category according the meat species tested (sheepmeat or beef).

| **No. of participants** | n |  |  |  |  | |  | |  |
| --- | --- | --- | --- | --- | --- | --- | --- | --- | --- |
| No. sheepmeat consumers | 39 |  |  |  |  | |  |  | |
| No. beef consumers | 36 |  |  |  |  | |  |  | |
| **Sheep taster - Gender** | **Men** | **Women** |  |  |  | |  |  | |
| No. sheepmeat consumers | 16 | 23 |  |  |  | |  |  | |
| No. beef consumers | 17 | 19 |  |  |  | |  |  | |
| **Age group (yrs.)** | **18.19** | **20-25** | **26-30** | **31-39** | **40-60** | **61-70** | |  | |
| No. sheepmeat consumers | 2 | 2 | 7 | 4 | 21 | 3 | |  | |
| No. beef consumers | 4 | 5 | 2 | 5 | 15 | 5 | |  | |
| **Sheep taster- cultural heritage** | **Australian** | **British descent** | **European descent** | **Asian descent** | **Other** | **Prefer not to say** | |  | |
| No. sheepmeat consumers | 20 | 7 | 3 | 5 | 3 | 1 | |  | |
| No. beef consumers | 24 | 5 | 0 | 5 | 1 | 1 | |  | |
| **Income (AUD per annum)** | **<50 K** | **50-70 K** | **70-90 K** | **90-110 K** | **110-130 K** | **130 K +** | | **unreported** | |
| No. sheepmeat consumers | 5 | 4 | 6 | 2 | 7 | 14 | | 1 | |
| No. beef consumers | 5 | 7 | 2 | 7 | 2 | 12 | | 1 | |
| **No. adults in household** | **1** | **2** | **3** | **4** | **5** |  | |  | |
| No. sheepmeat consumers | 7 | 26 | 4 | 1 | 1 |  | |  | |
| No. beef consumers | 3 | 22 | 8 | 2 | 1 |  | |  | |
| **No. of children in the household** | **0** | **1** | **2** | **3** | **4** | **5** | | **6** | |
| No. sheepmeat consumers | 24 | 4 | 8 | 1 | 0 | 1 | | 0 | |
| No. beef consumers | 21 | 8 | 3 | 3 | 1 | 0 | | 0 | |

**Table D1.** Number of participants per demographic category according the meat species tested (sheepmeat or beef)

| **Frequency of red meat consumption** | **once a day** | **4-5 times a week** | **2-3 times a week** | **weekly** | | **monthly** | | **never eat meat** | |  |  |
| --- | --- | --- | --- | --- | --- | --- | --- | --- | --- | --- | --- |
| No. sheepmeat consumers | 3 | 9 | 14 | 10 | | 3 | | 0 | |  |  |
| No. beef consumers | 2 | 9 | 19 | 4 | | 2 | | 0 | |  |  |
| **Frequency of sheepmeat / beef consumption** | **once a day** | **4-5 times a week** | **2-3 times a week** | **weekly** | | **monthly** | | **never eat meat** | |  |  |
| No. sheepmeat consumers | 3 | 2 | 17 | 12 | | 4 | | 0 | |  |  |
| No. beef consumers | 1 | 3 | 13 | 14 | | 4 | | 1 | |  |  |
| **Importance of red meat in the diet** | **I enjoy red meat, it's an important part of my diet** | | **I like red meat well enough, it's a regular part of my diet** | | **I do eat some red meat although truthfully it wouldn't worry me if I didn't** | | **unreported** | |  |  |  |
| No. sheepmeat consumers | 18 | | 13 | | 7 | | 1 | |  |  |  |
| No. beef consumers | 18 | | 12 | | 4 | | 2 | |  |  |  |
| **Cooking preference** | **Blue** | **Rare** | **Medium/ Rare** | **Medium** | | **Medium / Well Done** | | **Well Done** | |  |  |
| No. sheepmeat consumers | 0 | 4 | 14 | 12 | | 3 | | 5 | |  |  |
| No. beef consumers | 0 | 1 | 19 | 9 | | 4 | | 2 | |  |  |
| **Religious preferences** | **Halal certified** | **Kosher certified** | **none** | **other** | | **unreported** | |  | |  |  |
| No. sheepmeat consumers | 0 | 8 | 29 | 0 | | 2 | |  | |  |  |
| No. beef consumers | 0 | 7 | 25 | 0 | | 4 | |  | |  |  |
| **Main purchaser** | **Yes** | **No** |  |  | |  | |  | |  |  |
| No. sheepmeat consumers | 28 | 10 |  |  | |  | |  | |  |  |
| No. beef consumers | 21 | 14 |  |  | |  | |  | |  |  |
